# Excavating FAIR Data: the Case of the Multicenter Animal Spinal Cord Injury Study (MASCIS), Blood Pressure, and Neuro-Recovery

**DOI:** 10.1007/s12021-021-09512-z

**Published:** 2021-03-02

**Authors:** Carlos A. Almeida, Abel Torres-Espin, J. Russell Huie, Dongming Sun, Linda J. Noble-Haeusslein, Wise Young, Michael S. Beattie, Jacqueline C. Bresnahan, Jessica L. Nielson, Adam R. Ferguson

**Affiliations:** 1 Department of Neurological Surgery, Weill Institute for Neurosciences, Brain and Spinal Injury Center, University of California San Francisco, San Francisco, CA USA; 2 W.M. Keck Center for Collaborative Neuroscience, Rutgers University, New Brunswick, NJ USA; 3 Department of Neurology, University of Texas, Austin, TX USA; 4 Department of Psychology, University of Texas, Austin, TX USA; 5 Department of Psychiatry and Behavioral Sciences, University of Minnesota, Minneapolis, MN USA; 6 Institute for Health Informatics, University of Minnesota, Minneapolis, MN USA; 7 San Francisco Veterans Affairs Health Care System, San Francisco, CA USA

**Keywords:** Data science, Metascience, Neurotrauma, Reproducibility, Spinal contusion, Motor recovery, Autonomic, Hemodynamics

## Abstract

Meta-analyses suggest that the published literature represents only a small minority of the total data collected in biomedical research, with most becoming ‘dark data’ unreported in the literature. Dark data is due to publication bias toward novel results that confirm investigator hypotheses and omission of data that do not. Publication bias contributes to scientific irreproducibility and failures in bench-to-bedside translation. Sharing dark data by making it Findable, Accessible, Interoperable, and Reusable (FAIR) may reduce the burden of irreproducible science by increasing transparency and support data-driven discoveries beyond the lifecycle of the original study. We illustrate feasibility of dark data sharing by recovering original raw data from the Multicenter Animal Spinal Cord Injury Study (MASCIS), an NIH-funded multi-site preclinical drug trial conducted in the 1990s that tested efficacy of several therapies after a spinal cord injury (SCI). The original drug treatments did not produce clear positive results and MASCIS data were stored in boxes for more than two decades. The goal of the present study was to independently confirm published machine learning findings that perioperative blood pressure is a major predictor of SCI neuromotor outcome (Nielson et al., 2015). We recovered, digitized, and curated the data from 1125 rats from MASCIS. Analyses indicated that high perioperative blood pressure at the time of SCI is associated with poorer health and worse neuromotor outcomes in more severe SCI, whereas low perioperative blood pressure is associated with poorer health and worse neuromotor outcome in moderate SCI. These findings confirm and expand prior results that a narrow window of blood-pressure control optimizes outcome, and demonstrate the value of recovering dark data for assessing reproducibility of findings with implications for precision therapeutic approaches.

**Supplementary Information:**

The online version contains supplementary material available at 10.1007/s12021-021-09512-z.

## Introduction

The current system of biomedical research has generated enormous gains in knowledge, helping improve health outcomes over the past century. However, meta-analyses focusing on the practice of science have identified shortcomings in scholarly communications that limit the full potential of biomedical research. Estimates suggest that only 50% of completed clinical and preclinical studies are reported in the published literature (Chan et al., 2014). In addition, up to 85% of all biomedical research investment in data collection fails to yield publications, equating to a loss of over $200 billion in research investment worldwide per year (Chalmers & Glasziou, 2009; Røttingen et al., 2013). A consequence of failure to publish is “dark data”, where large quantities of research data remain locked away in hard-drives and file cabinets in formats difficult to access by the public or other interested parties (CMAJ, 2014). Furthermore, the published literature often reflects summaries of methods, protocols, and experimental results (e.g., *p* values, means, standard errors, graphs), which are not as informative as granular subject-level data used to derive these statistics (Chan et al., 2014). Making dark data accessible would improve the return on research investment by granting more people access to re-analyze and explore scientific data (Ferguson et al., 2014).

To improve value of biomedical research investment, Mueck (2013) and Wilkinson, et al. (2016) proposed making raw biomedical research data Findable, Accessible, Interoperable, and Reusable (FAIR). The FAIR data stewardship principles have been endorsed by the US National Institutes of Health (NIH) and major publishers. A major source of dark data are small granular data sets collected by laboratories over the course of day-to-day research, so called “long-tail data” (Ferguson et al., 2014). Long-tail data contain useful information such as non-targeted endpoints of experiments, alternative measures, and pilot data. In addition, long-tail data include results from failed experiments and ancillary records to published studies that were never published or disseminated. In animal research, such dark data often are recorded in veterinary care logs that are not considered primary endpoints in biomedical experiments. Recent efforts to collect and analyze dark data using advanced machine learning have yielded new findings with clinical implications (Hawryluk et al., 2015; Hawryluk et al., 2020; Nielson et al., 2015; Readdy et al., 2016). Specifically, by applying modern machine intelligence tools to archived data we discovered that mean arterial blood pressure (MAP) in the perioperative phase of SCI is a robust predictor of neuromotor recovery (Nielson et al., 2015).

This initial MAP finding relied on data recovered from one center from the multicenter animal spinal cord injury study (MASCIS), a preclinical drug trial conducted in the 1990s to compliment the National Acute SCI Study (NASCIS) human clinical trials comparing several experimental therapies against the anti-inflammatory glucocorticoid methylprednisolone in thoracic SCI. MASCIS had an enormous impact on the spinal cord injury (SCI) field. The consortium developed and validated the NYU-Impactor device to model contusive SCI (Constantini & Young, 1994), and standardized a locomotor outcome scale for rats (Basso et al., 1995; Basso et al., 1996). Both the NYU-Impactor and BBB locomotor scale remain widely used throughout preclinical SCI research (Young, 2002). However, the results of the treatment effects in MASCIS were never published.

The goals of the present project were to recover these dark data and make them FAIR, and to perform a multicenter replication/cross-validation of the previous single-center, machine-learning discovery that MAP predicted neuromotor outcome (Nielson et al., 2015). MASCIS data from the Ohio State University was previously used as our hypothesis generation dataset, where the finding about the negative impact of perioperative hypertension on SCI outcomes was discovered using a novel form of machine intelligence called topological data analysis (TDA) (Nielson et al., 2015). In the present study we used data from the remaining 7 sites as external cross-validation data to test the reproducibility of this hypothesis using traditional, confirmatory analytics.

Our team worked with original MASCIS consortium members to recover additional multicenter preclinical data collected across the study sites. After assembling a larger, and more representative MASCIS dataset from recovered paper records, we tested whether the Nielson et al. (2015) finding could be independently replicated using recovered data from the other MASCIS sites. Concurrent with this publication, we are releasing the recovered MASCIS data as a citable dataset (doi:10.34945/F5QG66) through the newly formed Open Data Commons for SCI (http://ODC-SCI.org), a public data sharing infrastructure (Callahan et al., 2017; Fouad et al., 2020). This serves our two adjacent purposes: providing meaningful scientific contributions to the field by cross-validating a clinically relevant finding, and converting MASCIS dark data and the millions of dollars spent on their acquisition (NIH R01 NS032000) into FAIR data that can continue to fuel new discoveries for SCI research into the future (Wilkinson et al., 2016).

## Methods

### MASCIS Data

Between 1993 and 1997, the NIH funded a consortium of eight laboratories (Wise Young, contact PI) to validate and standardize the MASCIS/NYU Impactor device used to give rats contusive SCI (Constantini & Young, 1994), and test promising treatments in a rat model for thoracic SCI. There were three studies in MASCIS. In 1994, the MP94 study assessed the effects of methylprednisolone (MP) on graded rat SCI across 3 injury severities (12.5, 25 and 50 mm weight drop contusions). The second study, in 1995 (YM95), assessed the effects of thyrotropin releasing hormone analogue YM14673 on the same SCI models. Both MP and YM14673 had been shown to improve recovery following a SCI (Behrmann et al., 1994; Constantini & Young, 1994; Faden, 1989). The third study (MY96) compared MP94 and YM95 protocols from the preceding years that the consortium determined were most successful. All centers executed the same methods and protocols, and all was approved by each institution’s respective Institutional Animal Care and Use Committee. The centers were: Ohio State University (Center 1), University of California - San Francisco (Center 3), Alfred I. DuPont Institute, Georgetown University Medical Center, Medical University of South Carolina, New York University, University of Florida - Gainesville, and Washington University School of Medicine (note we were not given explicit permission to re-identify these centers, but this information is available upon request). The protocols established for MP94 remained relatively unchanged until MY96. A notable exception was the exclusion of the 50 mm injury severity, which was too severe to reliably measure recovery. Because we were not able to recover data enough data from YM95, our current study focused on the data collected in MP94 and MY96.

### MASCIS Animals

Briefly, adult rats (age 77 ± 2 days) were randomly assigned to a graded contusion severity condition of either a 12.5, 25, or 50 mm weight drop for MP94, and only 12.5 and 25 mm for MY96 at thoracic level 9–10 (T9–10). Animals were assigned at random to a treatment group (MP94, Supplemental Table 1; MY96, Supplemental Table 2). All groups included equal number of males and females, and animal assigned to different contusion severity conditions. Perioperative systolic and diastolic blood pressure was monitored after the animal was anesthetized during the contusion surgery using an arterial catheter. The perioperative blood pressure values were recorded three different times during the procedure: within 20 min before to the moment of the SCI; at the moment of injury which was distinguished by a sharp spike in the blood pressure recording; and within the 20 min after the injury. Rats assigned to the acute survival condition were euthanized 48 h post SCI, and those in the chronic survival condition were evaluated using the Basso-Beattie-Bresnahan (BBB) locomotor scale (Basso et al., 1995; Basso et al., 1996) 2 days post SCI, and once per week for 6 weeks. All data collection were performed under institutionally approved animal care and use committee protocols at the constituent sites, adhering to federal standards.

### Legacy Data Retrieval

**Fig. 1:**
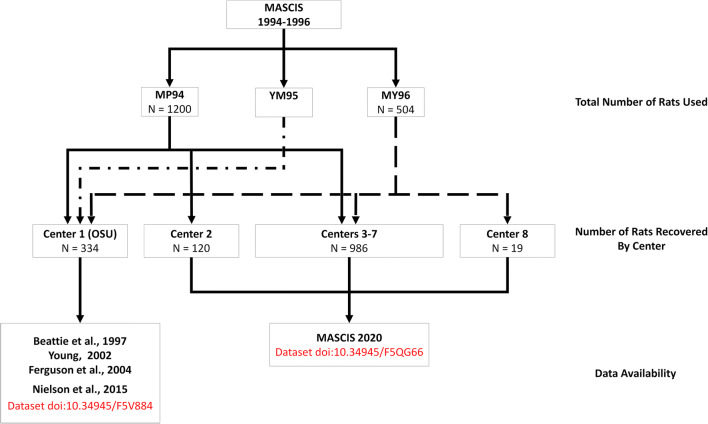
This flow chart describes the number of rats used in each of the three MASCIS studies, the number of rats for which data was recovered from each MASCIS study represented by solid or dashed lines, and where that data can be retrieved. An unknown number of rats were used in YM95. Data from Center 1 (OSU) were used in Beattie et al., 1997, Young 2002; Ferguson et al., 2004, and Nielson et al., 2015. The current paper describes data recovered from Centers 2–8, collectively titled MASCIS 2020. Note that Center 2 did not contribute data in MY96, and Center 8 only contributed data in MY96. The compiled dataset can be retrieved in odc-sci.org and is titled “ODC-SCI MASCIS”. Upon request or with permission, some centers can be unmasked

Our team worked with the original MASCIS team to track down the multicenter preclinical data collected by MASCIS across all sites. We learned that during the trials, copies of all data sheets from all centers, including surgery records, outcome measures, notes, etc., were sent to NYU (the primary center for MASCIS) to be analyzed. The treatment protocols in the study did not return significant findings, and the results of the treatment effects were never published. Shortly after MASCIS was concluded in 1997, the PI moved from New York University to Rutgers University and all data sheets, hard disks, computers, reports, protocols, and study materials were boxed up into 3 full sized moving trucks and stored in a commercial storage unit in Piscataway, New Jersey in a large storage unit (Neff, 2018). Our team of data archeologists gained access to the storage unit to search for MASCIS data from all study sites over two afternoons. We retrieved a few floppy disks that putatively contain records from the study, but we only had partial success in retrieving these data due to a combination of format obsolescence and ‘bit rot’ that occurs as magnetic media ages. In addition, we retrieved thousands of paper records which were scanned and converted to PDFs (Neff, 2018). The paper records were manually curated and organized into spreadsheet files, as had been done with the first iteration of Ohio State University data curated in the VISION-SCI repository (Nielson et al., 2014). Based on the results reported in the current paper (Fig. 1) we can surmise that the storage unit contains additional records buried within it or that data are lost to bit rot given the discrepancies in the intended sample size and the recovered sample size. That said, we have little reason to presume that the recovered data are not a representative sample of the population level effects.

### Data Entry and Curation

After studying original documents, protocols, and data sheets, we created a digital data template to digitize the data we recovered. When appropriate, we matched data fields from the hard copy data sheets to common data elements (CDEs) used by SCI data repositories (Nielson et al., 2014). Unique data fields were created for variables that were not CDEs.

As the data was digitized and the dataset populated, the goal was to enter the data as it was originally collected and written. However, some curation took place during data entry by fixing simple errors made by the original data creators. This level of curation required little field expertise, and involved transforming data to its intended form. For example, if the original protocol requested an animal’s temperature to be written in Celsius and the original data creator wrote the temperature in Fahrenheit, we converted those value back to Celsius. Another example was transforming values from milligrams (mg) to micrograms (μg) when the protocol and data sheet were intended for the data to be entered as micrograms. We also correct grammatical and spelling errors made by the original data creator when appropriate (e.g., then vs. than grammatical errors).

Though rarely employed, occasionally we omitted data if it could not be accurately recovered, and this required some domain expertise. Some information was lost or became illegible over the years, or when records were scanned for digitization. In these cases, it was inappropriate to guess the original data, and our team opted to leave those data points empty and considered them ‘missing’ in subsequent analysis. For example, if an animal’s weekly BBB scores read 2, 9, 11, 10, 12, 3, 11, the score second to last score was unexpected. While it is possible the second to last score was 3, it was also possible information was lost. It was inappropriate to fill in the expected value or guess the original score, but also inappropriate to disregard the legible value because it could be an outlier. In these situations we opted to consider the data missing in attempt to maintain data integrity and harnessed formal missing values analysis and robust methods to make statistical inferences in the face of missingness (see *statistical analysis*).

After we digitized all of paper records, we began our post data entry curation by looking at the mean, median, mode, minimum, and max values to identify errors in our database. For example, if the average temperature for a data field was 36.2 degrees Celsius and the mode is 35.9 degrees Celsius, but the max is value was 381 and the minimum was 3.72, the curator could fix those mistakes by assuming those were mistakes made during the digitizing phase when the dataset was being populated. When dealing with variables that required field expertise or when the curator was unsure if there was a mistake (e.g., anesthesia drug dose), the curator checked the values on the original data record. When that was not an option, or when our team was unsure about the quality of the data point after checking original records, we opted to leave the data point empty and consider the data point missing.

### VISION-SCI Data

Previously attempts to recover MASCIS data were included in the Visualized Syndromic Information and Outcomes for Neurotrauma-SCI (VISION-SCI) database funded by the National Institute of Neurological Disorders and Stroke (NINDS) to create a data repository by collecting retrospective data from animal models of SCI (Ferguson et al., 2011, 2013; Nielson et al., 2014). VISION-SCI retrieved subject-level data of approximatively 3000 mice, rats, and monkeys from 13 different laboratories from studies unpublished and published between 1993 and 2013. Part of the data incorporated into VISION-SCI came the Ohio State site in the Multicenter Animal Spinal Cord Injury Study (MASCIS) and was reported in Nielson et al., 2014. For the purposes of the current paper, these prior data from Nielson et al., 2014–2015 were excluded from analysis to reflect an independent replication of the results with subjects from non-OSU sites.

### Statistical Analyses

Our analysis was performed on the dataset we created from the paper records recovered. After digitizing and curating the records, data was analyzed using SPSS v25 (IBM Chicago, IL) and the statistical programing language R v3.6.0 (R Foundation for Statistical Computing, Vienna, Austria) with R Studio integrated development environment (R Core Team, 2019; RStudio Team, 2018). Missing values analysis was run using SPSS, and the null hypothesis that values were missing completely at random (MCAR) was tested using Little’s MCAR test.

Our MASCIS dataset included weekly values for BBB and weight. Some animals had multiple scores per week, and an aggregated score was calculated in those cases. We were interested in the effects of time measured in days post SCI, perioperative blood pressure, sex, and contusion drop height, on BBB locomotor recovery and weight gain. Using systolic and diastolic blood pressure values collected during SCI surgery, we estimated the perioperative mean arterial blood pressure values (MAP = (SBP + 2*DBP) / 3). Weight gain was calculated as the percent of change in weight (∆%weight) from baseline weight (e.g., a 30 g weight gain by a 300 g animal is 10% gain; ∆%weight = [weight / baseline weight] – 1 * 100). Locomotor recovery was the change in BBB score (∆BBB) calculated by subtracting the subject’s initial BBB score, recorded in the first 3 days post SCI, from the final BBB score before the subject expired or was perfused. A previous study has demonstrated BBB score does not significantly improve 22 days post SCI (Hook et al., 2004), so the final BBB score for a subject was used to calculate the change in BBB as long as locomotor evaluation took place 25 days post SCI.

We tested 4 separate Linear Mixed Models (LMM). Models were generated using lmer function in the R package lme4 (Bates et al., 2014), and the lmerTest package generated the Type III Analysis of Variance Table with Satterthwaite’s method (Kuznetsova et al., 2017). In the first pair of LMMs, we assessed BBB locomotor recovery after SCI as the outcome variable. Pre-injury blood pressure (MAP) collected within 20 min of SCI was a fixed factor the first LMM, and blood pressure (MAP) at time of injury (distinguished by a sharp spike in the blood pressure recording) was a fixed factor in the second LMM. In addition to pre-injury or at-injury blood pressure, time (days post SCI) and contusion severity (drop height) were fixed factors. Center and subject with a random slope by time were the random factor. For the third and fourth LMMs, we assessed weight gain after SCI as the outcome variable, as weight after injury is frequently used as a general measure of health and wellbeing. Pre-injury blood pressure (MAP) was a fixed factor the third LMM, and at-injury blood pressure (MAP) was a fixed factor in the fourth LMM. In addition to pre-injury or at-injury blood pressure, time (days post SCI) and contusion severity (drop height) were fixed factors. Center, sex, and subject with a random slope by time were the random factor. To explore interactions effects from the LMMs, we applied general linear models (GLM) using the lm function in R. Eta squared values were generated using the sjstats package in R (Lüdecke, 2020) or computed by custom code.

## Results

### Data Provenance and Descriptive Statistics on Recovered Data

According to the original MASCIS protocol, 1200 rats were planned for inclusion in 1994, including experiments to validate treatment protocols, anesthesia, and outcome measures (Supplemental Table 1)(Fig. 1). We recovered records from *n* = 252 rats with 2 days post SCI survival (Acute protocol), and *n* = 489 rats with 6 weeks post SCI survival (Chronic protocol). Records were recovered for an additional *n* = 31 animals, but we were unable to determine with certainty the intended survival time. In sum, we recovered records for 772 rats from MP94. The MASCIS 1996 protocol designated *n* = 504 rats for inclusion, and we recovered data for *n* = 353 of them (Supplemental Table 2). Prior work reported on 132 rats from MP94 and 72 from MY96, from the OSU cohort (Center 1) was described and accounted for in Nielson et al. (2015). We have excluded these data from analyses in the current work, but are making these dark data FAIR and releasing them as a companion to the current paper (doi:10.34945/F5QG66). Assuming all planned animals were included in the experiments and excluding animals from OSU, our recovery rate for MASCIS 1994 was 72.28% of animals, and 81.71% of animals for MASCIS 1996 (Fig. 1). There are 296 rats unaccounted for from MP94, 79 from MY96, and hundreds from YM95. During the data archeology expedition, we were not able to search all boxes in the storage unit, and the unaccounted for records may still be in storage. Moreover, some centers may not have sent copies of all their records to NYU before the study ended (e.g., in MASCIS 1996 internal progress reports, Center 4 contributed 11 of 72 records from the planned subject count, and Center 8 contributed 0 of 72 planned subject counts), which may partially explain why the number of animals per center in our dataset is not evenly distributed.

**Fig. 2:**
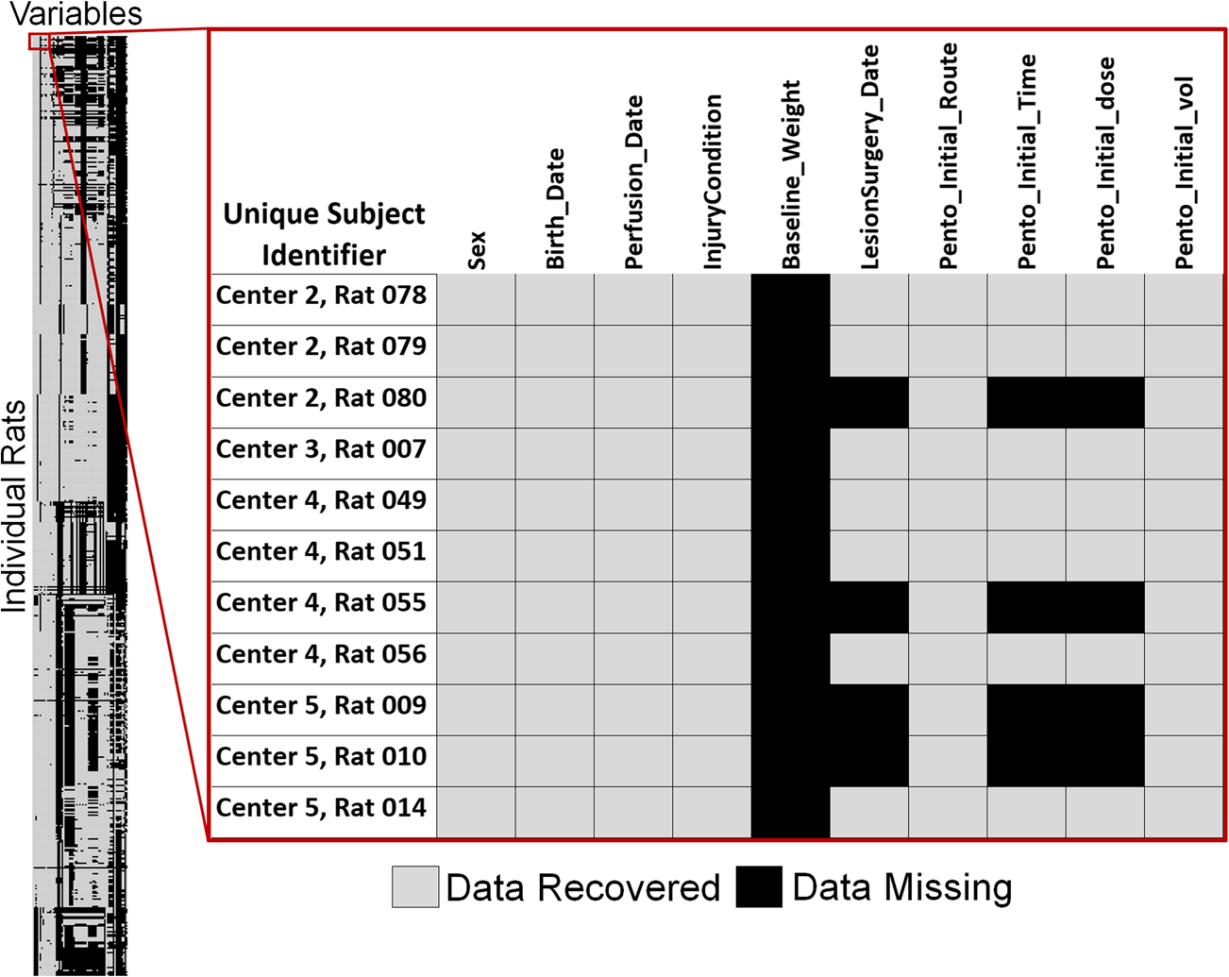
This heatmap demonstrates data recovered from the surgery record sheets of MP94 and MY96. Each row represents a unique rat (*n* = 1125), and each column represents a unique variable from the surgery records (*n* = 64), and each individual square a data point

Of the 1125 rats in our recovered dataset from MASCIS 1994 and MASCIS 1996, none have data records that were complete both within a test date and across all possible repeated measures (time-points). It is not always clear when post-operative records ended because perfusion dates were not always recorded in the perfusion logs we scanned. However, we were able to estimate our overall data recovery rate based on surgical records, which describes the subject’s surgery and condition for the first 48 h post injury. Surgery record sheets had 64 primary variables (Supplemental Table 3). Of the 1125 rats, *n* = 1121 had surgery records with at least 1 of the 64 variables completed. Our overall data recovery rate for surgery records was 60.44% (Fig. 2). This value might under-estimate recoverable data. Some rats died within 48 h of injury, while others were excluded from the study for reasons noted in their surgery sheets including anesthesia dosage, or surgery complications. For these reasons, we suspect portions of some surgery records were blank on purpose. We are confident that *n* = 500 survived postoperative complications because we recovered at least one data point collected at least 48 h post SCI from perfusion or post-operative care records. For those n = 500 rats, our surgery related data recovery rate was slightly better at 64.63%. Our objective is not to revisit data collection practices, nor compare data collection between centers. However, a data-driven missing values analysis demonstrated that values were not missing completely at random (Little’s MCAR test, *p* < 0.01).

Although we recovered substantial information from MASCIS, including original protocol and internal progress reports, we were not able to decipher the drug treatment blinding codes, and thus we cannot report on the results of the drug treatment protocol at the current time. Treatments were spread evenly between centers, animal sex, and contusion severity. According to the MASCIS progress reports, none of the MASCIS 1994 treatments resulted in better outcomes compared to control, and some treatments may have resulted in worse recovery and possibly death. The methylprednisolone 1 treatment in MASCIS 1996 resulted in better BBB recovery compared to saline. Moreover, according to unpublished MASCIS progress reports submitted to NIH, the independent variable that had the largest effect size on outcome was center. This opens the possibility that nuisance variables associated with specific centers drive the majority of the variance in outcome, potentially occluding drug effects. Our prior work strongly suggests that uncontrolled variance in operative blood pressure may be one such variable (Nielson et al., 2015).

### Confirmatory Hypothesis Testing of the Blood Pressure-Locomotor Recovery Association

We used LMMs to test the relationship between perioperative (20 min pre-injury and at-injury) blood pressure and BBB recovery, marshaling all available data recovered for each analysis while mitigating missing values (Nielson et al., 2020).

**Fig. 3:**
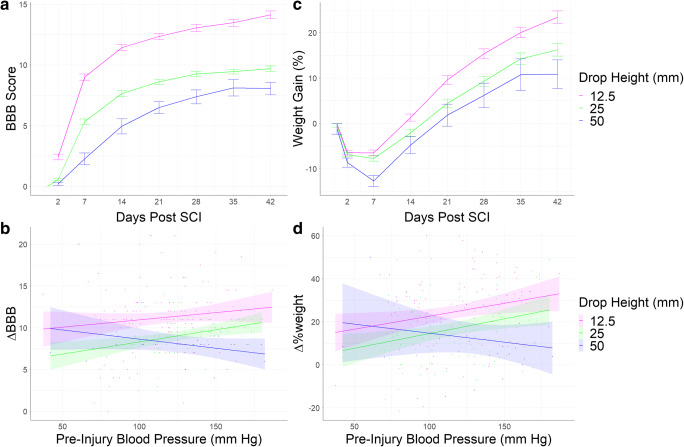
Change in BBB score (a) and weight gain (c) over time are shown with SEM bars for each time point. The linear relations between pre-injury blood pressure and ∆BBB (b) and ∆%weight (d) depicted, and the shaded areas represents the 95% confidence interval

The first LMM targeted pre-injury MAP as a predictor of BBB locomotor scores using 2327 observations from 441 unique rats across 6 Centers in the (Table 1; Fig. 3a). We found significant main effects for contusion severity and time on BBB scores. Animals with more severe contusions had worse BBB scores, and BBB scores improved as recovery time increased. There was also a significant three-way interaction between the pre-injury MAP, time post SCI, and contusion severity on BBB scores. This indicates that pre-injury blood pressure correlated with recovery of function, but this effect had different directionality depending on injury severity.

**Table 1:** Linear Mixed Model Output of BBB with Pre-Injury Blood Pressure as Fixed Factor

Variables	NumDf	DenDf	F-Value	p value	*η*^2^
Pre-Injury Blood Pressure	1	506.38	1.5174	0.2186	0.002
Contusion Severity	2	435.62	8.6503	0.0002	0.038
Days Post SCI	1	788.47	147.1760	<0.0001	0.157
Pre-Injury Blood Pressure x Contusion Severity	2	430.87	0.0242	0.9761	<0.001
Pre-Injury Blood Pressure x Days Post SCI	1	771.89	0.5725	0.4495	<0.001
Pre-Injury Blood Pressure x Contusion Severity x Days Post SCI	2	743.54	9.7646	<0.0001	0.025

In the second LMM targeted at-injury MAP as a predictor of BBB locomotor outcome scores using 1081 observations among 197 unique rats across 4 Centers (Table 2). There were significant main effect for contusion severity and time on BBB scores. Locomotor scores improved as recovery time increased, and scores decreased as contusion severity increased. There was a significant two-way interaction between at-injury MAP and contusion severity, and a three-way interaction between at-injury MAP, time post SCI, and contusion severity on BBB scores.

**Table 2:** Linear Mixed Model Output of BBB with Blood Pressure at SCI as Fixed Factor

Variables	NumDf	DenDf	F-Value	p value	*η*^2^
At-Injury Blood Pressure	1	398.70	0.1556	0.6935	<0.001
Contusion Severity	2	208.55	8.9549	0.0002	0.079
Days Post SCI	1	119.22	39.2645	<0.0001	0.247
At-Injury Blood Pressure x Contusion Severity	2	220.07	3.9490	0.0207	0.034
At-Injury Blood Pressure x Days Post SCI	1	118.61	0.0972	0.7557	<0.001
At-Injury Blood Pressure x Contusion Severity x Days Post SCI	2	125.52	9.8191	0.0001	0.135

Post hoc analyses were required to understand the precise nature of the significant blood pressure-recovery interactions uncovered by LMM analyses. We used a GLM to assess the effect of pre-injury MAP and contusion severity on ∆BBB from baseline to the time of the rat’s expiration (Table 3). As shown in Fig. 3b, for moderate SCI (12.5, 25 mm weight-drop), higher pre-injury MAP associated with better outcome, whereas for severe SCI (50 mm) higher MAP associated with worse outcomes. This indicates that pre-injury blood pressure affected recovery of function, and this relationship had different directionality depending on injury severity, with more severe SCI demonstrating a more profound negative influence of high blood pressure. The same form of effect was observed with at-injury MAP, suggesting that perioperative blood pressure is a robust predictor of BBB.

**Table 3:** General Linear Model Comparing ∆BBB Across Groups

Variables	Df	F-Value	p value	*η*^2^
Pre-Injury Blood Pressure	1	1.4429	0.2309	0.0050
Contusion Severity	2	19.6133	<0.0001	0.1420
Pre-Injury Blood Pressure x Contusion Severity	2	3.1911	0.0430	0.0230
Residual	230			

The third LMM targeted the pre-injury MAP on weight gain using 2336 unique observations among 414 rats across 5 Centers (Table 4; Fig. 3c). There were significant main effect for pre-injury MAP and time on ∆%weight, and significant two-way interactions between pre-injury MAP and time and pre-injury MAP and contusion severity on ∆%weight. Lastly, we found a significant three-way interaction between contusion severity, time post injury, and pre-injury MAP on ∆%weight gain.

**Table 4:** Linear Mixed Model Output of Weight Gain with Pre-Injury Blood Pressure as Fixed Factor

Variables	NumDf	DenDf	F-Value	p value	*η*^2^
Pre-Injury Blood Pressure	1	393.72	5.4686	0.0199	0.013
Contusion Severity	2	406.60	2.0256	0.1332	0.009
Days Post SCI	1	272.51	8.8732	0.0032	0.031
Pre-Injury Blood Pressure x Contusion Severity	2	405.77	2.9243	0.0548	0.014
Pre-Injury Blood Pressure x Days Post SCI	1	268.60	21.4986	<0.0001	0.074
Pre-Injury Blood Pressure x Contusion Severity x Days Post SCI	2	263.72	11.6871	<0.0001	0.081

The fourth LMM included 1349 unique observations between 276 rats across 3 Centers (Table 5). In this model, we found a significant main effect of time on ∆%weight, and a significant three-way interaction between at-injury MAP, contusion severity, and time on ∆%weight.

**Table 5:** Linear Mixed Model Output of Weight Gain with Blood Pressure at SCI as Fixed Factor

Variables	NumDf	DenDf	F-Value	p value	*η*^2^
At-Injury Blood Pressure	1	284.93	0.5197	0.4716	0.002
Contusion Severity	2	255.31	1.1172	0.3288	0.008
Days Post SCI	1	118.83	24.0393	<0.0001	0.168
At-Injury Blood Pressure x Contusion Severity	2	248.99	1.0228	0.3611	0.008
At-Injury Blood Pressure x Days Post SCI	1	117.75	2.3158	0.1307	0.019
At-Injury Blood Pressure x Contusion Severity by Days Post SCI	2	129.89	20.2185	<0.0001	0.237

Post hoc GLM was required to further understand the effects on ∆%weight. This analysis revealed a marginally significant interaction effect between pre-injury MAP and contusion severity (Table 6). For animals in the low and medium contusion severity conditions, higher blood pressure was associated with more weight recovery. The inverse was true for the high contusion severity group, where higher blood pressure was associated with less weight recovery (Fig. 3d). The analysis of weight change also showed that males recovered and gained more weight compared to females, but there were no significant interaction effects that included the sex of the animal, suggesting the interaction between perioperative blood pressure and contusion severity is not significantly different between males and females.

**Table 6:** General Linear Model Comparing ∆%weight Across Groups

Variables	Df	F-Value	p value	*η*^2^
Pre-Injury Blood Pressure	1	10.0654	0.0017	0.025
Contusion Severity	2	16.5195	<0.0001	0.083
Sex	1	132.8158	<0.0001	0.335
Pre-Injury Blood Pressure x Contusion	2	2.7582	0.0657	0.014
Pre-Injury Blood Pressure x Sex	1	0.0989	0.7535	<0.001
Contusion x Sex	2	0.2040	0.8156	0.001
Pre-Injury Blood Pressure x Contusion x Sex	2	0.6046	0.5472	0.003
Residual	213			

We did not find meaningful results when we included post-injury MAP as fixed factors in our LMMs (results not shown), and this analysis is confounded by known effects of injury severity on subsequent autonomic derangements (i.e., potential for associations reflecting ‘reverse causality’ with SCI severity) (Nout et al., 2012). Altogether, the results suggest that perioperative *hyper*tension is associated with poorer health and worse locomotor recovery in more severe SCI whereas perioperative *hypo*tension is associated with poorer health and worse recovery in moderate SCI.

## Discussion

In the current confirmatory study, we recovered legacy data from 1125 rats to independently replicate the results from Nielson et al. (2015). Our results suggest an interaction effect between perioperative blood pressure and contusion severity, where rats with more severe injuries and higher blood pressure had less recovery, while rats with milder injuries and higher blood pressure showed better recovery. To our knowledge, this is the first time such an interaction between blood pressure, injury severity, and recovery has been demonstrated in cases of SCI. In achieving our goal of cross validating a prior finding of high clinical import, we recovered value from the initial millions of dollars of investment by the NIH made over two decades ago in the original MASCIS trials, and demonstrated a practical application FAIR data principles (Table 7).

**Table 7:** FAIR data principles checklist for BPM replication in MASCIS

Principle	Definition	Compliance
F – FINDABLE	**To be Findable:** F1. (meta)data are assigned a globally unique and persistent identifier F2. data are described with rich metadata (defined by R1 below) F3. metadata clearly and explicitly include the identifier of the data it describes F4. (meta)data are registered or indexed in a searchable resource	Original MASCIS data F1 – yes F2 – no F3 – yes F4 – no MASCIS data entered into VISION-SCI/ODC-SCI F1 – yes F2 – yes F3 – yes F4 – yes
A – ACCESSIBLE	**To be Accessible:** A1. (meta)data are retrievable by their identifier using a standardized communications protocol A1.1 the protocol is open, free, and universally implementable A1.2 the protocol allows for an authentication and authorization procedure, where necessary A2. metadata are accessible, even when the data are no longer available	Original MASCIS data A1 – maybe A1.1 – no A1.2 – N/A (unknown) A2 – maybe (in the protocols/grants?) MASCIS data entered into VISION-SCI/ODC-SCI A1 – yes A1.1 – yes A1.2 – yes A2 – yes
I – INTEROPERABLE	**To be Interoperable:** I1. (meta)data use a formal, accessible, shared, and broadly applicable language for knowledge representation. I2. (meta)data use vocabularies that follow FAIR principles I3. (meta)data include qualified references to other (meta)data	Original MASCIS data I1 – no I2 – no I3 – no MASCIS data entered into VISION-SCI/ODC-SCI I1 – yes I2 – yes I3 – yes
R - REUSABLE	**To be Reusable:** R1. meta(data) are richly described with a plurality of accurate and relevant attributes R1.1. (meta)data are released with a clear and accessible data usage license R1.2. (meta)data are associated with detailed provenance R1.3. (meta)data meet domain-relevant community standards	Original MASCIS data R1 – no R1.1 – no R1.2 – yes R1.3 – yes MASCIS data entered into VISION-SCI/ODC-SCI R1 – yes R1.1 – yes R1.2 – yes R1.3 – yes

In addition, the results have direct implications for clinical care in acute SCI. Managing MAP in acute SCI may be critically important for preventing secondary injuries and neurological deficits. In the published guidelines for acute medical and surgical management of SCI, the American Association of Neurological Surgeons (AANS) and Congress of Neurological Surgeons (CNS) supported maintenance of MAP above 85 and 90 mmHg for patients during the first week after admission (Hadley et al., 2002; Walters et al., 2013; Yue et al., 2017). The rationale is that low blood pressure reduces blood flow and patients that are kept at a higher MAP after SCI show better recovery (Casha & Christie, 2011; Catapano et al., 2016; Dakson et al., 2017; Hawryluk et al., 2015; Sabit et al., 2018). Nielson et al. (2015) were the first to note that hypertension, in addition to hypotension, impairs recovery.

One of the important implications of our findings pertains to precision medicine. Kepler et al. (2015) reported that patients with pre-existing hypertension had worse recovery compared to controls. They proposed that blood pressure goals for those patients may have to be set even higher than those recommended by AANS and CNS, and further studies are needed to identify the role of hypertension, blood flow to the spinal cord, and recovery (Kepler et al., 2015). We agree that such studies are needed, due to the lack of consensus in clinical protocol guidelines for maximum blood pressure for patients after SCI. The Center for Disease Control and Prevention estimates one in three Americans are hypertensive (Center for Disease Control and Prevention, 2020), and data is needed to identify MAP goals that would maintain tissue function without impairing the neurological recovery for that population. Recent retrospective clinical studies of high-resolution physiological monitoring further supports MAP should be maintained above 85–90 mmHg up to seven days upon the patient’s admission to a hospital, and the proportion of time below 85 mmHg correlated with impaired recovery (Hawryluk et al., 2015; Sabit et al., 2018; Walters et al., 2013). Physiologically, the rational is that spinal cord perfusion pressure depends on systemic MAP remaining high enough to sustain tissue oxygenation in the injury penumbra in the face of vertebral fracture and cord compression (Squair et al., 2019; Yue et al., 2020). This SCI clinical guideline mirrors the logic of intracranial pressure monitoring in traumatic brain injury and other fields of cranial neurosurgery where prevention of hypotension using fluids and vasopressors is used to maintain intracranial pressure and decompressive hemicraniectomy is used to prevent pressure overshoot (Chesnut et al., 2020; Shah et al., 2019). However, the concept of hypertension as a driver of poor outcome is less well established. In the wake of Nielson et al., 2015 several clinical groups have begun exploring hypertension as a potential negative prognosticator of outcome. The first of these was recently published, in the form of a case series providing preliminary clinical support for the hypothesis (Ehsanian et al., 2020). Physiologically, it would stand to reason that hypertension may result in ‘hemorrhagic conversion’, and exacerbate bleeding into the spinal cord and resulting in tissue damage. In the animal literature it is well established that SCI compromises the blood-spinal-cord barrier and that peripheral blood components contribute to secondary cell death, including infiltration of circulating immune cells, circulating cytokines and other factors (Crowe et al., 1997; Ferguson et al., 2008; Kigerl et al., 2009).

The major limitation in our analysis is that it is correlational, and not causal. In addition, our conclusions come from incomplete retrospective data. Not all of the original data was recovered, and some may be permanently lost due format obsolescence and bit rot of magnetic media. Not included in the data recovered were the drug treatment codes. The rats were treated with various drugs, and we remain blinded to their treatment condition. According to the MASCIS progress reports, all but one treatment condition did not show a significant recovery associated with treatment. However, this does not rule out the possibility that specific dose-response and timing features for methylprednisolone and other tested drugs may have impacted the results. In addition, variation in animal care may also have introduced confounds. For example, MASCIS used the anesthetic pentobarbital, which is known to produce blood pressure complications (Nout et al., 2012). Some centers closely monitored blood oxygenation and performed resuscitation as needed, whereas other centers were less focused on these anesthetic complications. In addition, post-operative care protocols evolved over time, especially with respect to bladder care and antibiotic use to control mortality due to urinary tract infections. One of the centers discovered the fluoroquinolone Baytril was highly effective at reducing post-SCI mortality, and this was later adopted by the other centers. Accordingly, analyses by the original MASCIS consortium determined the independent variable that affected outcomes most was center, and data recovered was not evenly distributed across centers. This suggests that there is substantial variability in healthcare records, even in a well-controlled and protocolized randomized control trial (RCT) in animal subjects which have greater standardization of housing, diet, health care and study conditions than a human RCT. The fact that large center effects persist even under these idealized conditions may be due to the fact that randomization is performed and monitored in a small number of indexed variables and may not apply to non-indexed variables such as high blood pressure in MASCIS. Whether center-to-center variability is less in animals versus human RCTs, or controlled trials versus observational is an interesting open question that FAIR data sharing may help resolve in the future. Making individual participant data FAIR could enable translational cross-walk meta-analysis between humans and animals, if privacy and security concerns that arise from multidimensional clinical data can be appropriately mitigated (Rocher et al., 2019). Although we statistically controlled for the effect center in the present paper, and confirmed blood pressure effects, this post-hoc statistical approach is less powerful than a balanced prospective study for inferring causal relationships. We therefore recommend a prospective study assessing the impact of hypertension on recovery after SCI of different severities, where center and treatment differences can be more directly controlled for.

Neurological trauma and related disorders are incredibly complicated to treat. Due to the complexity and heterogeneity of SCI and central nervous system (CNS) disorders, our viewpoint is that researchers would benefit by approaching these diseases as ‘big-data’ problem, specifically involving big data *variety* (Ferguson et al., 2011; Hawkins et al., 2019; Huie et al., 2018). SCI may result in motor control and mobility impairments; impaired breathing and respiratory deficits; loss of bladder function; bowel and sexual dysfunctions; pathological pain; and/or loss of autonomy. To capture the multivariate syndromic outcomes of CNS disorders, researchers often collect multiple outcome measures for each individual subject. However, outcomes are often only assessed a few factors at a time. Complex and contemporary analytical methods, including those more easily associated with –omics, which permit researchers to explore the multi-dimensionality of diseases rather than testing a few factors at a time are becoming increasingly more accessible and common in biomedicine (Parikshak et al., 2015), and as was the case with Nielson et al. (2015) these methods will continue drive future biomedical research. Accelerating the transition from a univariate to a multivariate view of diseases should be a target for biomedicine, and making data FAIR through data sharing and data archeology are crucial and achievable steps in making that transition (Callahan et al., 2017; Ferguson et al., 2011, 2013; Fouad et al., 2020).

While there are reservations about data sharing among classically trained biology researchers, the –omics science disciplines have successfully navigated those concerns for decades (Kaye et al., 2009; Lander, 1996). Genomic and other –omic published studies always provide the accession number to National Center for Biotechnology Information (NCBI) datasets used for their analyses, where all datasets are publicly available for download. In addition, many authors make their codes and scripts publicly available on platforms like GitHub (GitHub Inc., San Francisco, CA) for anybody to replicate and validate their analysis. While this may be a novel concept for some, members of neurotrauma disciplines have established pathways for data sharing (Fisher et al., 2009; Fouad et al., 2020; Huerta et al.,s 1993; Lemmon et al., 2014; Marmarou et al., 2007).

Despite limitations, our study shows that even legacy data from 25 years ago may yield important findings, and this helps support emerging standards that all NIH funded research should follow FAIR data stewardship principles (Mueck, 2013; Wilkinson et al., 2016). The first attempt to gather subject-level data from neurotrauma studies was VISION-SCI (Nielson et al., 2014), but to our knowledge the present work represents the first targeted attempt of data retrieval of animal subject level data at this scale. The MASCIS consortium was a large and expensive group with a budget that exceeded $1 million annually between 1994 and 1996, and used over 2000 animals for their experiments. Our inability to recover the original treatment conditions for rats from MASCIS is not unusual given the regulatory standards under which these data were collected. For the majority of grant funded research, historically, NIH mandated that data be maintained for 3–5 years post-study completion (NIH Office of Extramural Research, 2019). Having retrieved data for over 1000 animals at an estimated data recovery rate above 60%, our experience retrieving part of that dataset was overall successful because we increased the retained value from the original investment. Additionally, we are adding these data to our prior recovered data from OSU in our public release of the MASCIS data as part of this paper yielding a total of 1459 animals data records made FAIR through data archeology.

While data archeology may increase the initial investment in some circumstances, such as those presented here from the MASCIS study, we strongly recommend and endorse pursuing a policy of applying FAIR data principles for neuroscience as data are collected, and specifically making raw subject level data accessible to the greater scientific community. Efforts to incorporate this into study designs at the onset of data collection would ensure FAIR data access moving forward. While it is unclear whether data archeology is as laborious as prospective data collection, the scientific community risks losing data if is not collected and disseminated adherent to FAIR principles as we demonstrated in this project. The NIH and NINDS CDEs greatly facilitate the opportunities of researchers sharing data among collaborators or colleagues, and new platforms to facilitating data sharing already exist or will soon be available for many disciplines in biomedical research (Hawkins et al., 2019).

The present work extends the concept of meta-analysis to raw source data, which opens new possibilities to develop higher evidence for preclinical studies (currently classed as level 4–5 evidence) (Biering-Sørensen, 2005). At the current time it remains unclear to what extent systematic reviews and meta-analyses can be relied upon to be correct reflections of raw data (Gøtzsche et al., 2007) as they are based primarily on statistics reported in papers. In addition, reviews suggest that over 80% of published manuscripts in a biomedical science journal contains a least one statistical error (Simundic & Nikolac, 2009), and there are no indications that statistical rigor is increasing in biomedical research (Ercan, 2015; Ercan et al., 2017). Thus, there is much to be gained by granting the next generation of scientist’s access to FAIR datasets derived by data archeology, data recovery, and application of modern data stewardship and analytic tools of the sort applied here.

## Information Sharing Statement

Our analysis code reproducing our figures and results are available as part of the supplementary material online. All data collated from MP94 and MY96 are available at: doi:10.34945/F5QG66.

## Supplementary Information

ESM 1 (DOCX 25 kb)
