# Supplementary material for: Excavating FAIR Data: the Case of the Multicenter Animal Spinal Cord Injury Study (MASCIS), Blood Pressure, and Neuro-Recovery
Source: Neuroinformatics. 2021 Mar 2;20(1):39–52. doi: 10.1007/s12021-021-09512-z (PMC9015816; doi:10.1007/s12021-021-09512-z)
Supplement: Supplementary file 1 — (DOCX 25 kb) [file 12021_2021_9512_MOESM1_ESM.docx]

| **Supplemental Table 1.** List of MP94 Experiments and Treatments | | |
| --- | --- | --- |
| Experiment | n | Description |
| Pre-randomize | 253 | Impact parameters and lesion volumes in untreated rats / Validation experiments |
| Anesthesia | 92 | Intravenous vs. intraperitoneal pentobarbital experiments |
| OFT Validation | 150 | Multicenter OFT validation and inter-rater analysis |
| Delayed MP | 108 | Compare 30, 120, 240, and 360 minutes MP on 48h lesion |
|  | 152 | Compare -10 (pre), 30, 60, vs. 120 minutes MP on 24h lesion |
| Acute MP | 245 | Randomize +30 vs. +60 vs. +120 minute initiation time, 24h vs. 48h, and 30 vs. 60 mg/kg MP therapy |
| Chronic MP | 200 | Randomized +30 vs. +60 vs. +120 minute initiation time, 24h vs. 48h, and 30 vs. 60 mg/kg MP therapy |
| Total | 1200 |  |

| **Supplemental Table 2.** List of MY96 Treatments | | |
| --- | --- | --- |
| Treatment | n | Protocol Description |
| MP1 | 72 | MP 30 mg/kg @ 5 min & Sal 0.5 ml/h @ 1h • 23 |
| MPC | 72 | MP 30 mg/kg @ 5 min & MP 5.4 ml/kg/h @ 1h • 23 |
| MPY | 72 | MP 30 mg/kg @ 5 min & YM 1.0 mg/kg @ 3h & 0.1 ml/kg/h @ 3h • 20 |
| Veh | 72 | Sal 0.5 ml @ 5 min & Sal 0.5 ml/hr @ 1h • 23 |
| YME | 72 | YM 1.0 mg/kg @ 5 min & YM 0.1 mg/kg/hr @ 1h • 20 |
| YML | 72 | Sal 0.5 ml @ 5 min, YM 1.0 mg/kg @ 3h & YM 0.1 mg/kg/h @ 3h • 20 |
| Total | 504 |  |
|  |  |  |

| Supplemental Table 3. List of Variables in Surgery Sheet and Percent of Data Recovered for Variables | | | | |
| --- | --- | --- | --- | --- |
| Variable | **% Recovered** | **Variable** | **% Recovered** | |
| Treatment Set | 94.04 | At Hit Diastolic Max | | 36.00 |
| Treatment Box | 93.07 | At Hit Diastolic Min | | 35.56 |
| Treatment Rat | 77.78 | At Hit Systolic Max | | 36.27 |
| Sex | 99.47 | At Hit Systolic Min | | 35.73 |
| Date of Birth | 67.64 | Post Inj. Time | | 82.93 |
| Date of Expiration | 89.96 | Post Inj. pH | | 74.58 |
| Contusion Drop Height | 98.40 | Post Inj. PCO2 | | 70.76 |
| Pre-Op Weight | 98.22 | Post Inj. O2 | | 70.58 |
| Date of Operation | 98.67 | Post Inj. HCO3 | | 51.02 |
| Surg. Anes. Drug | 98.49 | Post Inj. Base E | | 41.07 |
| Surg. Anes. Drug Route | 98.93 | Post Inj. O2 Sat | | 47.91 |
| Surg. Anes. Drug Time | 96.71 | Post Inj. Temp | | 80.27 |
| Surg. Anes. Drug Dose | 78.40 | Post Inj. Diastolic Max | | 80.36 |
| Surg. Anes. Drug Volume | 87.82 | Post Inj. Diastolic Min | | 71.82 |
| Time of Injury | 95.73 | Post Inj. Systolic Max | | 80.89 |
| Surg. Antibiotic Drug | 73.07 | Post Inj. Systolic Min | | 72.71 |
| Surg. Antibiotic Drug Route | 53.16 | Treatment Drug Code | | 79.11 |
| Surg. Antibiotic Drug Time | 34.22 | Treatment Dose Volume | | 87.47 |
| Surg. Antibiotic Drug Dose | 30.93 | Perfusion Time | | 42.04 |
| Surg. Antibiotic Drug Volume | 52.27 | Perfusion Anes. Drug | | 36.00 |
| Pre Inj. Time | 84.09 | Perfusion Anes. Route | | 40.71 |
| Pre Inj. pH | 69.78 | Perfusion Anes. Dose | | 33.33 |
| Pre Inj. PCO2 | 66.84 | Perfusion Anes. Volume | | 14.84 |
| Pre Inj. O2 | 66.58 | Perfusion Weight | | 63.02 |
| Pre Inj. HCO3 | 43.47 | Perfusion Hematology Bilirubin | | 8.89 |
| Pre Inj. Base E | 33.07 | Perfusion Hematology Urea | | 8.53 |
| Pre Inj. O2 Sat | 40.18 | Perfusion Autopsy Samples Blood | | 5.69 |
| Pre Inj. Temp | 76.98 | Perfusion Autopsy Samples Plasma | | 7.91 |
| Pre Inj. Diastolic Max | 80.36 | Perfusion Autopsy Samples Urine | | 18.22 |
| Pre Inj. Diastolic Min | 71.56 | Perfusion Autopsy Samples Spinal | | 19.38 |
| Pre Inj. Systolic Max | 81.16 | Perfusion Autopsy Finding | | 2.76 |
| Pre Inj. Systolic Min | 72.27 | Perfusion Autopsy Notes | | 37.33 |
